# A Single-Center (Sibiu, Romania), Retrospective Study (March–November 2020) of COVID-19 Clinical and Epidemiological Features in Children

**DOI:** 10.3390/jcm10163517

**Published:** 2021-08-10

**Authors:** Maria Totan, Felicia Gabriela Gligor, Lavinia Duică, Nicolae Grigore, Sinziana Silișteanu, Ionela Maniu, Elisabeta Antonescu

**Affiliations:** 1 Faculty of Medicine, Lucian Blaga University of Sibiu, 2A Lucian Blaga Str., 550169 Sibiu, Romania; felicia.gligor@ulbsibiu.ro (F.G.G.); lavinia.duica@ulbsibiu.ro (L.D.); nicolae.grigore@ulbsibiu.ro (N.G.); elisabeta.antonescu@ulbsibiu.ro (E.A.); 2 Clinical Laboratory, Clinical Pediatric Hospital, 2-4 Pompeiu Onofreiu Str., 550166 Sibiu, Romania; 3 County Clinical Emergency Hospital, 2-4 Corneliu Coposu Str., 550245 Sibiu, Romania; 4 Department of Health and Human Development, Stefan cel Mare University of Suceava, 13 University Str., 720229 Suceava, Romania; sinziana.silisteanu@usm.ro; 5 Research Team, Pediatric Clinical Hospital Sibiu, 550166 Sibiu, Romania; 6 Research Center in Informatics and Information Technology, Mathematics and Informatics Department, Faculty of Sciences, “Lucian Blaga” University of Sibiu, 550025 Sibiu, Romania

**Keywords:** coronavirus, COVID-19, SARS-CoV-2, children, pediatric, clinical features, epidemiology

## Abstract

The aim of this study was to describe and analyze epidemiological and clinical features of children screened for COVID-19 at Sibiu Pediatric Clinical Hospital during the first 9 months (March–November) of coronavirus disease pandemic in Romania. A total of 203 pediatric patients with a confirmed diagnosis of COVID-19 were included in the study. The median age of the patients was 121 (IQR 18–181) months and 52.22% had mild clinical type with pneumonia, 35.47% were moderate cases, 3.94% severe cases, 0.99% critically ill cases and 7.39% were asymptomatic. The most common symptoms were fever (n = 130, 64.03%), nasal congestion (n = 138, 67.98%), cough (n = 128, 63.05%) followed by sore throat (n = 64, 31.52%), rhinorrhea (n = 63, 31.03%), fatigue (n = 57, 28.07%), headache (n = 47, 23.15%), diarrhea (n = 39, 19.21%), vomiting (n = 32, 15.76%), myalgia (n = 24, 11.82%), abdominal pain (n = 22, 10.83%). A higher proportion of infants with severe or critical disease was encountered with lymphopenia (n = 9, 90%), neutrophilia (n = 5, 50%), leukocytosis (n = 5, 50%) compared with asymptomatic infants (n = 10, 66.67%, n = 1, 6.67%, n = 3, 20%) or mild (n = 53, 50%, n = 19, 17.92%, n = 15, 14.15%) and moderate (n = 37, 51.39%, n = 9, 12.50%, n = 6, 8.33%) cases (*p* = 0.095, *p* = 0.042, *p* = 0.034). Pediatric patients generally had mild or moderate type of COVID-19, and the critically ill cases were rare. In our study, frequent symptoms were observed in both the systemic and respiratory systems, ear, nose and throat system, and less from gastrointestinal system, central nervous system or ocular system. Additionally, there is an increase in liver and myocardial enzyme levels with an increase in disease severity. Understanding the clinical and laboratory characteristics of pediatric patients is important for diagnosis, management and effective control of the disease.

## 1. Introduction

SARS-COV-2 (severe acute respiratory syndrome coronavirus 2) virus is a new type of coronavirus and was identified as the cause of an outbreak of pneumonia in China in late 2019 [1,2,3,4]. The epidemic has spread worldwide despite efforts to limit it [2,5,6,7,8].

The main route of transmission of the virus is the respiratory tract, through the respiratory droplets, the incubation period being between 5 and 14 days [1]. Symptomatology of the disease is predominantly respiratory (fever, cough, difficulty breathing) [9,10]. The vast majority of cases present symptoms of minimal and moderate intensity, and only in about 20% of cases may occur severe manifestations (bilateral interstitial pneumonia), with evolution to respiratory failure and acute respiratory distress (ARDS). There are also cases with gastrointestinal manifestations (especially diarrhea), and in some patients, anosmia (loss of smell) and dysgeusia (loss of taste) have been reported as early symptoms. The reference test for the specific diagnosis of COVID-19 (coronavirus disease 2019) is a molecular biology test, by NAAT technology (nucleic acid amplification technology). This test is reverse transcription real-time PCR (qRT-PCR) which highlights the presence of viral RNA in the nasopharynx [11]. 

Studies on clinical features and prognosis of pediatric patients are fewer in children compared to adults, and they indicate a much milder clinical course and severity of COVID-19 in children [12,13,14].

Studies reporting the results of screening for COVID-19 underlying clinical and epidemiological factors denote that most children appear to have mild or moderate disease or many non-specific symptoms or may by asymptomatic [13,15,16,17,18,19,20,21].

The aim of this study is to describe and analyze epidemiological and clinical features of children screened for COVID-19 at Sibiu Pediatric Clinical Hospital. This information could contribute to extend the understanding of this disease in pediatric patients.

## 2. Materials and Methods

This was a retrospective, single-center study, including all children under 18 years confirmed with SARS-CoV-2 infection, diagnosed by RT-PCR, tested between 1 March 2020 and 30 November 2020, hospitalized in the Pediatric Clinical Hospital, Sibiu. This study was approved by the ethics committee of the Pediatric Children’s Hospital Sibiu (no. 4065/12 June 2020) and followed the Declaration of Helsinki. The diagnosis of COVID-19 was based on the criterion of the World Health Organization (WHO) interim guidance [22].

The data were obtained retrospectively from electronic medical records. We reviewed demographic data, exposure history, clinical symptoms, mixed infections, comorbidities, laboratory data, radiological characteristics, treatment and outcomes. Exposure history was focused on the source of infection (SARS-CoV-2 infection diagnosis in the child’s family, close contacts with a possible/confirmed SARS-CoV-2-infected individual). Clinical symptoms include the symptoms recorded at the time of hospitalization/testing. Mixed infections were defined as the concurrent infection of a patient with 2 or more pathogens.

The included clinical types of COVID-19 in pediatric patients were defined as asymptomatic infection, mild, moderate, severe and critical cases, based on the clinical features, laboratory testing and chest X-ray imaging [15,16,17,18].

1. Asymptomatic infection: without any clinical symptoms, signs and abnormal chest imaging at time of screening but with positive RT-PCR test for SARS-CoV-2.
2. Mild: with upper respiratory symptoms (fever, fatigue, myalgia, cough, sore throat, nasal congestion, runny nose, digestive symptoms, etc.) and without abnormal radiography or auscultatory abnormalities.
3. Moderate: with pneumonia and symptoms such as fever, cough, nasal congestion, sore throat, fatigue, headache and with abnormal radiography.
4. Severe: mild or moderate clinical features accompanied by: oxygen saturation less than 92%, lack of consciousness/convulsions and any other manifestations suggesting injuries to vital organs.
5. Critical: rapid disease progression, respiratory failure with need for mechanical ventilation, organ failure monitoring in the intensive care unit.

Laboratory examinations included hematological, serum biochemical (measurement of biomarkers for monitoring liver, myocardial, and renal functions), and acute-phase protein tests. We considered laboratory references as normal ranges adjusted to age and gender, as defined in the “Reference ranges for adults and children: pre-analytical considerations” [23].

RT-PCR tests were conducted to identify the presence of the virus in the respiratory tract by collecting a nasal exudate and a pharyngeal exudate on the first day of hospitalization. For RT-PCR testing, automatic extraction is used, on a 24-position extractor Lab-Aid 824-Zeesan, and the amplification is done on a BIORAD CFX2s device.

We presented categorical variables as frequencies and percentages while continuous variables were presented as mean (SD: standard deviation) when values were normally distributed and median (IQR: interquartile range) otherwise. Comparison between groups were conducted using Chi-square or Fisher’s exact test in case of categorical data and independent group t-tests, ANOVA, Mann–Whitney U or Kruskal–Wallis H test in case of continuous data. A *p*-value <0.05 was considered statistically significant.

## 3. Results

RT-PCR was applied to 1719 children who attended the Pediatric Clinical Hospital, Sibiu, between 1 March 2020 and 30 November 2020. Of these, 203 patients representing confirmed COVID-19 cases (positive for the RT-PCR test) were included in the study. The median age was 121 (IQR 18–181) months and female cases were predominant (n = 106, 52.22 % vs. n = 94, 47.78%).

The sources of infection were by close contact with family members (n = 131, 64.53%), contact with other suspected cases (n = 24, 11.82%), and 48 patients (23.65%) were cases with unidentified source of infection. Thirty family clusters involving 71 children were encountered. Family clusters were defined as 3 or more than 3 people (at least 2 children and at least 1 adult family member) with confirmed cases in one family. These were mostly secondary cases, where the transmission was from adults to children.

Patients in our study presented mild (n = 106, 52.22%), moderate (n = 72, 35.47%), severe (n = 8, 3.94%), critically ill disease (n = 2, 0.99%), and 15 (7.39%) patients were asymptomatic. We considered asymptomatic patients as children who presented to the hospital for other medical problems (limb fractures, appendicitis, etc.) that required testing and were thus diagnosed with SARS-CoV-2 infection.

Demographic data and clinical characteristics of cases are summarized in Table 1.

On admission, the most frequent symptoms were fever (n = 130, 64.03%), nasal congestion (n = 138, 67.98%), cough (n = 128, 63.05%), followed by sore throat (n = 64, 31.52%), rhinorrhea (n = 63, 31.03%), fatigue (n = 57, 28.07%), headache (n = 47, 23.15%), diarrhea (n=39, 19.21%), vomiting (n = 32, 15.76%), myalgia (n = 24, 11.82%), abdominal pain (n = 22, 10.83%), with a median of 4 (IQR 3-5) for symptoms/patient.

Some symptoms differed between mild and moderate clinical type of COVID-19. In cases of children with moderate clinical type, there were more children with sore throat (n = 34, 47.22% vs. n = 30, 28.30%, *p* = 0.010), headache (n = 25, 34.72% vs. n = 21, 19.81%, *p* = 0.026), rhinorrhea (n = 29, 40.28% vs. n = 32, 30.19%, *p* = 0.164), fatigue (n = 26, 36.11% vs. n = 28, 26.42%, *p* = 0.167) than in case of mild clinical type. There was no significant difference in fever (n = 52, 72.22% vs. n = 73, 68.87%, *p* = 0.631) and cough (n = 51, 70.83% vs. n = 75, 71.75%, *p* = 0.991) between moderate and mild groups. Other respiratory co-infection with candida (n = 78, 32.42%) and hemolytic staphylococcus aureus (n = 37, 18.22%) were reported. Some patients also presented comorbidities like anemia (n = 41, 20.19%), malnutrition (n = 16, 7.88%), epilepsy (n = 7, 3.45%) and renal failure (n = 2, 0.99%). In cases of children with gastrointestinal manifestations, no other significant findings (adenovirus, rotavirus, norovirus) were observed in routine stool tests. Abnormal radiographic presentations were seen in 40.39% (n = 82) of cases, 18.23% (n = 37) pediatric patients had pulmonary ground-glass opacities, and 22.17% (n = 45) had patchy shadows. Unilateral lesions were encountered in less than 5% of patients (n = 9, 4.43%) and bilateral lesions were detected in 35.96% (n = 73) of pediatric patients.

The laboratory examination related to hematological, serum biochemical (measurement of biomarkers for monitoring liver, myocardial, and renal functions), and acute-phase protein are reported in Table 2.

On laboratory assessment, 53% of patients were noted to have lymphopenia, neutropenia 32.02% (n = 65), and low iron 23.37% (n = 43). In contrast, patients also had monocytosis (n = 90, 44.33%), elevated C-reactive protein (n = 51, 25.12%, a significant inflammatory marker [24] also in COVID-19), neutrophilia (n = 34, 16.75%), leukocytosis (n = 29, 14.29%), elevated iron (n = 22, 11.96%), and increased number of eosinophils (n = 24, 11.82%).

Biomarkers for monitoring liver, myocardial and renal functions revealed high level of alanine aminotransferase in 8.87% (n = 18) of cases, high level of aspartate transferase in 9.85% (n = 20) of cases, and high level of creatinine in 2.96% (n = 6) of cases while there was only one case with low values for each of these biomarkers. There were 6.40% (n = 13) cases with low-level values of urea and only 3 (1.48%) patients had high level of urea.

The association test (Chi-square or Fisher) results indicated that there are significant differences in values of leucocyte, neutrophil and lymphocyte between considered clinical types. A higher proportion of infants with severe or critical disease was encountered with lymphopenia (n = 9, 90%), neutrophilia (n = 5, 50%), leukocytosis (n = 5, 50%) compared with asymptomatic infants (n=10, 66.67%, n = 1, 6.67%, n = 3, 20%) or mild (n = 53, 50%, n = 19, 17.92%, n = 15, 14.15%) and moderate (n = 37, 51.39%, n = 9, 12.50%, n = 6, 8.33%) cases (*p* = 0.095, *p* = 0.042, *p* = 0.034).

Most patients had a mild disease progression and received only antipyretics treatment. Hydroxychloroquine and lopinavir-ritonavir syrup were administered to 23.15% (n = 47) patients, antibiotics were received by 38.42% (n = 78), and mechanical ventilation was required for 2 (0.99%) patients. Twelve patients received antibiotic therapy due to concerns about viral-bacterial mixed co-infection. Additionally, antibiotics were given to five patients who were diagnosed with other acute upper respiratory tract infections before detection and confirmation of COVID-19. All patients were discharged except the two cases with critical ill condition who died. The median duration of hospitalization was 4 (IQR 2–9) days for all patients. The length of stay in mild (5.50, IQR 2–11) and moderate (4, IQR 3–7) cases was shorter than in severe (8.50, IQR 4–13) cases (Table 3). In some cases, the hospitalization was shorter than the duration of viral shedding. These cases were quarantined for 2 weeks at home, in order to prevent the spread of the virus. Close contacts were mandatorily isolated for 14 days and were monitored by telephone by public health officials.

In this study, there were two cases of patients who died. These patients had several co-existing comorbidities (pre-existing medical conditions; neurological disorders). Previous reports also indicated a small proportion of critically unwell children and, as in adults, with chronic pre-existing comorbidities [3,25,26].

### 3.1. Case 1

An 11-month-old boy with a past history of epilepsy and major neonatal encephalopathy was admitted for respiratory distress with fever related to bilateral pneumonia. SARS-CoV-2 was identified on nasopharyngeal swabs by RT-PCR. Despite antibiotics, antivirals, and anticoagulation therapy, there was a rapid respiratory deterioration progressing to acute respiratory distress syndrome. He underwent intubation and noninvasive mechanical ventilation but developed refractory hypoxemia and multiorgan failure. He died 6 days after the confirmation of SARS-CoV-2.

### 3.2. Case 2

A 3-year and 11-month-old boy with a past history of autism, severe psychomotor impairment, flaccid paraparesis, and Holt–Oram syndrome. The disease started with fever (38.5 °C) and cough. Radiography was performed, and findings were typical of COVID-19 infection, which was confirmed by PCR (nasopharyngeal swabs). He underwent noninvasive high-flow nasal O_2_ therapy (20–25 L/min to 50 L/min, 40% SpO2). The results of imagistic reevaluation indicated unfavorable evolutionary aspects and appearance of abscessed pneumonia (with SpO2 38%). During the hospital stay, he received drugs, convalescent plasma therapy, and needed orotracheal intubation and mechanical ventilation. He developed severe bradycardia followed by asystole and did not respond to resuscitation maneuvers. He died 23 days after the confirmation of SARS-CoV-2.

## 4. Discussion

This study examines clinical and epidemiological characteristics of pediatric SARS-CoV-2 infection from Romania (most of the research articles on pediatrics patients include Chinese data [1,2,3,5,6,7,11,12,13,14,16,18,20,21,26]).

More than 50% of infected children had close contact with family members with COVID-19. Spread of the infection in patients’ families had also been reported in various studies [11,27,28].

The clinical manifestations of pediatric patients are predominantly asymptomatic or mild disease; critical diseases were seen only in two patients, in agreement with reported results in pediatric patients. These findings advocate that COVID-19, which has caused millions of deaths worldwide, has a better prognosis in children than adults [16,29,30,31,32]. Why this happens needs further investigation. Angiotensin 2 conversion enzyme (ACE2) has been established as a functional receptor for SARS-CoV-2 [33,34] and which provides viral entry into human cells [35,36]. ACE2 is encountered in a variety of cells from different human organs (lung alveolar epithelial, small intestinal epithelial, vascular endothelial, smooth muscle cells, brush border cells, cellular cells, parietal epithelial cells, basal epidermal layer of skin, oral and nasal mucosa) and absent in others (lymphoid tissular and hepatobiliary structures) but the role of ACE2-expressing organs do not equally participate in COVID-19 pathophysiology [33,35]. There are studies indicating that ACE2 maturity and function in children may be lower than in adults [37,38]. Additionally, children rarely have comorbidities (nephrotic syndrome, insufficient cardiac, hypertension, diabetes, liver diseases, and dyslipidemia) compared with adults.

In our study, frequent symptoms were observed in both the systemic and respiratory systems, ear, nose and throat system, and less from gastrointestinal system, central nervous system or ocular system. These results are consistent with those from previous reports. Symptoms such as fever and cough in more than 60% of cases were mentioned by [13,20,26]. In our study, episodes of fever as a symptom (mentioned at the time of hospitalization) were considered. Research in pediatric patients reported fever and cough as the most common symptoms in children diagnosed with COVID-19 with the proportion of fever between 20% and 100%, respectively, and proportion of cough between 11% and 100% [19,39,40,41,42]. Moreover, research in adult patients reported the fever and cough as the most common symptoms with prevalence of fever at 78% (95% CI 75–81) and prevalence of cough 57% (95% CI 54–60) [43]. A possible explanation of percentual variation may be related to (small) sample size and/or of how fever is defined: temperature measured at the time of admission or measured before or during hospitalization, different levels of temperature (>37 °C or >37.3 °C or >37.5 °C or >38 °C or >39 °C or unmentioned) [12,13,19,40,44]. In the present study, symptoms such as sore throat, rhinorrhea, fatigue, headache, diarrhea, vomiting, myalgia, and abdominal pain, were observed in a lower percentage of patients. These percentages are in accordance with some pediatric studies [20], and in contrast with others [19] or, are approximatively 10% higher than the estimates for adults [43].

Our results of abnormal laboratory findings indicated lymphopenia in more than 50% of cases. Moreover, in cases of severe or critically ill patients, 90% had lymphopenia. Our results are comparable with those of [18,45], but are in disagreement with [12,46,47,48,49]. With adult patients, lymphopenia was found in 82% of cases in [50], with values between 2.9% and 72.3% in the review [51].

Present results showed higher proportion of children with severe or critical disease with lymphopenia, neutrophilia, or leukocytosis compared with asymptomatic children with mild and moderate cases. In the review [52] including 24 studies with 270 participants with multisystem inflammatory condition (MIS-C), case studies from Europe and the United States, leukocytosis, neutrophilia and lymphopenia were encountered accompanied by one or more increased inflammatory markers (CRP, procalcitonin (PCT), erythrocyte sedimentation rate (ESR), ferritin, IL-6, D-Dimer). The main manifestations were fever and gastrointestinal symptoms (abdominal pain or vomiting). When selecting children with abdominal pain or vomiting in our study, the results were similar with [52] and a different profile was noticed in comparison with the whole group: leukocytosis 18.75% vs. 14.29%, neutrophilia 27.08% vs. 16.75%, lymphopenia 75% vs. 53.69%, elevated CRP 41.67% vs. 25.12% [52]. In a study in neonates and young infants [53], neutropenia and/or monocytosis without lymphopenia was detected in more than 70% of cases. Additionally, other studies associated lymphopenia with progression of lung disease [54,55,56]. This association between lymphopenia and progression of disease severity has also been noticed in our study, with 90% of cases from severe or critical group having lymphopenia compared with approximatively 50% from mild or moderate group (*p* = 0.006, *p* = 0.005).

According to our current data, there is an increase in levels of liver and myocardial enzymes with the increase of the disease severity (ALT: asymptomatic 16 (IQR 13–22) vs. mild 16.5 (IQR 12–26) vs. moderate 19 (IQR 13.5–30.5) vs. severe or critically ill 21 (IQR 16–23), AST: asymptomatic 24 (IQR 19–37) vs. mild 28 (IQR 20–39) vs. moderate 27 (IQR 20–43) vs. severe or critically ill 28.5 (IQR 20–45)) but the differences are not statistically significant as in another study [57] (ALT: mild 13 (IQR 11–18.8) vs. moderate 18 (IQR 12.3–33.8), AST: mild 25 (IQR 20.3–34.5) vs. moderate 33 (IQR 24–46.8)).

One limitation of this study is that it was a retrospective and single-center study of pediatric patients. The laboratory data includes only the markers which are usually recorded by any hospital. For a better understanding of COVID-19 disease in children, additional detailed patient data collected on a long-term follow-up (multicentered, if possible) could improve our findings. Further research including additional biomarkers (Immunoglobulins IgA, IgG, IgM, IgE; cytokines, tumor necrosis factor (TNF-α), D-Dimer; procalcitonin (PCT)) may help to (i) extend the understanding of the risk factors associated with SARS-CoV-2 severity and (ii) improve clinical management. Additionally, many recent research reports have shown particular aspects regarding number of platelets (thrombocytosis/thrombocytopenia), morphology of platelets and volume (hyperchromatic, protruding in pseudopodia formations) [58,59,60]. Future studies might envisage advanced laboratory techniques to predict increased level of risks for prothrombotic events using nanomedicine approaches such as AFM, FTIR, and RAMAN imaging [61,62,63,64]. In addition, analyzing samples other than blood, saliva for example, with its electrolyte imbalances for sodium, potassium, and calcium might show promise to differentiate mild and severe COVID-19 disease forms [65,66] in children. Moreover, laboratory parameters may have been influenced by other patient-related factors and preexisting health conditions, which we did not consider in the study for lack of information.

Comparison of clinical, hematological, and immunological profiles between children with COVID-19 pneumonia and/or healthy children and/or non-COVID-19 pneumonia, could also help in obtaining evidence about the role of different biomarkers in the development of COVID-19 pneumonia. As there were only two children with severe COVID-19 during the study period, we could not define the exact reasons for the severity of the disease or a profile for severe cases. However, our results indicated that children with pre-existing comorbidities (in our study, neurological disorders) have a predisposition to critical illness.

Our study offers insights into pediatric cases with COVID-19, diagnosed and treated in a Centre in Romania. Understanding the clinical and laboratory characteristics of pediatric patients is important for diagnosis, management, and effective control of the disease. Our results and information from existing reports could be used as clues in further researches.

## Figures and Tables

**Table 1:** Characteristics of patients on admission, stratified by clinical types.

Patient Characteristics		Total 203(100)	Asymptomatic 15(7.4)	Mild 106(52.2)	Moderate 72(35.5)	Severe 10(4.9)
**Gender**	Male	97(47.78)	10(66.67)	46(43.40)	34(47.22)	7(70.00)
	Female	106(52.22)	5(33.33)	60(56.60)	38(52.78)	3(30.00)
**Age**	Median mo. (IQR)	121 (18;181)	155 (84;168)	100.50 (14;176)	133.50 (25.50;187)	74.50 (25;168)
	<1 year	39(19.21)	0(0.00)	23(21.70)	14(19.44)	2(20.00)
	1–5 year	35(17.24)	2(13.33)	22(20.75)	8(11.11)	3(30.00)
	6–10 year	27(13.30)	4(26.67)	15(14.15)	7(9.72)	1(10.00)
	>10 year	102(50.25)	9(60.00)	46(43.40)	43(59.72)	4(40.00)
**Source of infection**	Contact with family members	131(64.53)	0(0.00)	76(71.70)	54(75.00)	1(10.00)
	Contact with suspected cases	24(11.82)	1(6.67)	10(9.43)	12(16.67)	1(10.00)
	Unidentified source of infection	48(23.65)	14(93.33)	20(18.87)	6(8.33)	8(80.00)
**Time from onset to hospital admission** **(median (IQR))**		2(1;4)		2(1;3)	2(1;4)	1(1;5)
**Radiography**	Unilateral	9(4.43)		0(0.00)	9(12.50)	0(0.00)
	Bilateral	73(35.96)		0(0.00)	63(87.50)	10(100)
	Ground-glass opacity	37(18.23)		0(0.00)	38(25.78)	7(70.00)
	Patchy shadows	45(22.17)		0(0.00)	34(47.22)	3(30.00)
**Symptoms**	Median no. of symptoms (IQR)	4 (3;5)		4 (3;5)	5 (3;6)	1.50 (1;3)
	Fever	130(64.03)		73(68.87)	52(72.22)	5(50.00)
	Fatigue	57(28.07)		28(26.42)	26(36.11)	3(30.00)
	Myalgia	24(11.82)		17(16.04)	6(8.33)	1(10.00)
	Cough	128(63.05)		75(70.75)	51(70.83)	2(20.00)
	Sore throat	64(31.52)		30(28.30)	34(47.22)	0(0.00)
	Rhinorrhea	63(31.03)		32(30.19)	29(40.28)	2(20.00)
	Nasal congestion	138(67.98)		77(72.64)	58(80.56)	3(30.00)
	Hyposmia/hypogeusia	20(9.85)		12(11.32)	7(9.72)	1(10.00)
	Diarrhea	39(19.21)		25(23.58)	13(18.06)	1(10.00)
	Vomiting	32(15.76)		18(16.98)	12(16.67)	2(20.00)
	Nausea	6(2.955)		5(4.72)	1(1.39)	0(0.00)
	Abdominal pain	22(10.83)		15(14.15)	7(9.72)	0(0.00)
	Headache	47(23.15)		21(19.81)	25(34.72)	1(10.00)
	Dizziness	4(1.97)		2(1.89)	2(2.78)	0(0.00)
**Mixed infections**	Candida	78(38.42)		41(38.68)	35(48.61)	2(20.00)
	Hemolytic staph. aureus	37(18.22)		14(13.21)	21(29.17)	2(20.00)
**Comorbidities**	Anemia	41(20.19)		22(20.75)	18(25.00)	1(10.00)
	Malnutrition	16(7.88)		7(6.60)	5(6.94)	4(40.00)
	Renal failure	2(0.99)		1(0.94)	0(0.00)	1(10.00)
	Epilepsy	7(3.45)		3(2.83)	0(0.00)	4(40.00)

**Table 2:** Laboratory findings of patients on admission, stratified by clinical types.

Laboratory Data		Total 203(100)	Asymptomatic 15(7.4)	Mild 106(52.2)	Moderate 72(35.5)	Severe 10(4.9)
**White blood cells** **cells/µL**	Median (IQR)	6500 (4850;8780)	7870 (5810;9760)	6645 (5160;9820)	5980 (5425;9425)	11,645 (5970;9530)
	Decreased	6(2.96)	0(0.00)	3(2.83)	3(4.17)	0(0.00)
	Increased	29(14.29)	3(20.00)	15(14.15)	6(8.33)	5(50.00)
**Neutrophils** %	Mean ± SD	44.09 ± 19.24	50.87 ± 16.01	43.16 ± 19.51	41.98 ± 18.45	58.89 ± 20.54
	Decreased	65(32.02)	4(26.67)	31(29.25)	29(40.28)	1(10.00)
	Increased	34(16.75)	1(6.67)	19(17.92)	9(12.50)	5(50.00)
**Lymphocytes** %	Mean ± SD	41.37 ± 18.33	36.41 ± 13.31	42.38 ± 18.86	43.09 ± 17.94	25.70 ± 15.03
	Decreased	109(53.69)	10(66.67)	53(50.00)	37(51.39)	9(90.00)
	Increased	27(13.30)	0(0.00)	13(12.26)	13(18.06)	1(10.00)
**NLR**	Median (IQR)	1.13 (0.59;1.96)	1.48 (1.04;1.95)	1.03 (0.50;1.98)	1.10 (0.62;1.66)	2.92 (1.20;4.82)
**Monocytes** %	Mean ± SD	12.05 ± 5.25	9.87 ± 3.91	11.78 ± 4.85	12.95 ± 5.91	11.60 ± 5.45
	Increased	90(44.33)	4(26.67)	45(42.45)	36(50.00)	5(50.00)
**Eosinophils** %	Median (IQR)	1.20 (0.40;2.40)	1.40 (0.60;4.00)	1.15 (0.40;2.50)	1.20 (0.20;2.05)	0.50 (0.10;1.80)
	Decreased	3(1.48)	0(0.00)	1(0.94)	1(1.39)	1(10.00)
	Increased	24(11.82)	3(20.00)	12(11.32)	7(9.72)	2(20.00)
**Basophils** **%**	Median (IQR)	0.30 (0.20;0.50)	0.30 (0.20;0.50)	0.30 (0.20;0.40)	0.30 (0.20;0.50)	0.30 (0.10;0.40)
	Decreased	1(0.50)	0(0.00)	1(0.94)	0(0.00)	0(0.00)
	Increased	6(2.97)	0(0.00)	3(2.86)	3(4.17)	0(0.00)
**C-reactive protein** **mg/L**	Median (IQR)	2.00 (2.00;5.70)	2.00 (2.00;3.15)	3.00 (2.00;9.00)	2.00 (2.00;3.00)	2.14 (2.00;3.00)
	Decreased	1(0.50)	0(0.00)	1(0.94)	0(0.00)	0(0.00)
	Increased	51(25.12)	2(13.33)	32(30.19)	16(22.22)	1(10.00)
**AST** **U/L**	Median (IQR)	27.00 (20.00;41.00)	24.00 (19.00;37.00)	28.00 (20.00;39.00)	27.00 (20.00;43.00)	28.50 (20.00;45.00)
	Decreased	1(0.50)	0(0.00)	1(0.94)	0(0.00)	0(0.00)
	Increased	20(9.85)	1(6.67)	11(10.38)	7(9.72)	1(10.00)
**ALT** **U/L**	Median (IQR)	17.00 (13.00;26.00)	16.00 (13.00;22.00)	16.50 (12.00;26.00)	19.00 (13.50;30.50)	21.00 (16.00;23.00)
	Decreased	1(0.50)	0(0.00)	1(0.94)	0(0.00)	0(0.00)
	Increased	18(8.87)	1(6.67)	8(7.55)	8(11.11)	1(10.00)
**Urea** **mg/dL**	Mean ± SD	23.55 ± 7.70	22.46 ± 3.96	23.32 ± 7.84	23.23 ± 7.47	29.90 ± 9.96
	Decreased	13(6.40)	0(0.00)	9(8.49)	4(5.56)	0(0.00)
	Increased	3(1.48)	0(0.00)	1(0.94)	1(1.39)	1(10.00)
**Creatinine** **mg/dL**	Mean ± SD	0.61 ± 0.17	0.63 ± 0.10	0.58 ± 0.17	0.64 ± 0.17	0.62 ± 0.20
	Decreased	1(0.50)	0(0.00)	1(0.94)	0(0.00)	0(0.00)
	Increased	6(2.96)	0(0.00)	3(2.83)	2(2.78)	1(10.00)
**Iron** **µmol/L**	Median (IQR)	9.10 (5.52;14.50)	10.20 (5.80;14.50)	8.80 (4.92;14.64)	9.12 (5.90;14.44)	5.93 (4.50;18.52)
	Decreased	43(23.37)	1(9.09)	24(25.00)	14(20.29)	4(50.00)
	Increased	22(11.96)	1(9.09)	10(10.42)	9(13.04)	2(25.00)

**Table 3:** Treatment and outcome of patients, stratified by clinical types.

Treatment and Outcome		Total 203(100)	Asymptomatic 15(7.4)	Mild 106(52.2)	Moderate 72(35.5)	Severe 10(4.9)
**Treatment**	Antibiotic therapy	78(38.42)	0(0.00)	31(29.25)	39(54.17)	8(80.00)
	Antiviral therapy	47(23.15)	0(0.00)	16(15.09)	26(36.11)	5(50.00)
	Mechanical ventilation	2(0.99)	0(0.00)	0(0.00)	0(0.00)	2(20.00)
**Duration of hospitalization** **(days, median (IQR))**		4 (2;9)	2 (2;4)	5.50 (2;11)	4 (3;7)	8.50 (4;13)
**Clinical outcome**	Discharged	201(99.01)	15(100.00)	106(100.00)	72(100.00)	8(80.00)
	Died	2(0.99)	0(0.00)	0(0.00)	0(0.00)	2(20.00)

## Data Availability

The data presented in this study are available on request from the principal author. The data are not publicly available due to privacy restrictions.
